# Stellate Cells from Rat Pancreas Are Stem Cells and Can Contribute to Liver Regeneration

**DOI:** 10.1371/journal.pone.0051878

**Published:** 2012-12-13

**Authors:** Claus Kordes, Iris Sawitza, Silke Götze, Dieter Häussinger

**Affiliations:** Clinic of Gastroenterology, Hepatology and Infectious Diseases, Heinrich Heine University Düsseldorf, Düsseldorf, Germany; Institute of Hepatology London, United Kingdom

## Abstract

The identity of pancreatic stem/progenitor cells is still under discussion. They were suggested to derive from the pancreatic ductal epithelium and/or islets. Here we report that rat pancreatic stellate cells (PSC), which are thought to contribute to pancreatic fibrosis, have stem cell characteristics. PSC reside in islets and between acini and display a gene expression pattern similar to umbilical cord blood stem cells and mesenchymal stem cells. Cytokine treatment of isolated PSC induced the expression of typical hepatocyte markers. The PSC-derived hepatocyte-like cells expressed endodermal proteins such as bile salt export pump along with the mesodermal protein vimentin. The transplantation of culture-activated PSC from enhanced green fluorescent protein-expressing rats into wild type rats after partial hepatectomy in the presence of 2-acetylaminofluorene revealed that PSC were able to reconstitute large areas of the host liver through differentiation into hepatocytes and cholangiocytes. This developmental fate of transplanted PSC was confirmed by fluorescence in situ hybridization of chromosome Y after gender-mismatched transplantation of male PSC into female rats. Transplanted PSC displayed long-lasting survival, whereas muscle fibroblasts were unable to integrate into the host liver. The differentiation potential of PSC was further verified by the transplantation of clonally expanded PSC. PSC clones maintained the expression of stellate cell and stem cell markers and preserved their differentiation potential, which indicated self-renewal potential of PSC. These findings demonstrate that PSC have stem cell characteristics and can contribute to the regeneration of injured organs through differentiation across tissue boundaries.

## Introduction

The rodent pancreas has the capacity to regenerate [1] and attempts were made to unravel the function of tissue specific stem cells in this process. There is still no consensus about the identity and origin of pancreatic stem/progenitor cells. They were suggested to derive from the pancreatic ductal epithelium and/or from pancreatic islets [2]–[6]. Recent findings suggest that vitamin A-storing hepatic stellate cells (HSC) exhibit features of stem and progenitor cells [7]. Stellate cells can be found as pericytes in many organs of vertebrates including the pancreas. These pancreatic stellate cells (PSC) are supposed to contribute to wound healing and fibrosis of the pancreas [8], [9], but little is known about their origin and function in normal pancreas. Isolated PSC develop into myofibroblast-like cells during culture on plastic and start to express α-smooth muscle actin (α-SMA) as well as extracellular matrix proteins. This culture-dependent activation of PSC was frequently used as a model to analyze molecular mechanisms of fibrogenesis *in vitro*. Quiescent stellate cells from liver and pancreas contain vitamin A mainly as retinyl palmitate, which is stored in characteristic lipid vesicles. PSC possess fewer lipid droplets and their vitamin A content is lower than in HSC [10]. The storage of retinoids is an established and useful marker to identify stellate cells. They can be further identified by their expression of filamentous proteins such as glial fibrillary acidic protein (GFAP), desmin, synemin and vimentin [8], [9], [11]–[14]. Nestin is another filamentous protein synthesized by activated stellate cells from liver and pancreas [15], [16]. Interestingly, nestin synthesis is elevated during activation of somatic stem cells [17], suggesting that PSC may represent undifferentiated cells. The aim of the present study was to investigate stem/progenitor cell characteristics of PSC with a special focus on the analysis of the *in vivo* differentiation potential of PSC into extrapancreatic cell types such as hepatocytes. The latter is a known feature of pancreas-specific stem cells [18]–[21], whose identity is still unclear.

**Figure 1:**
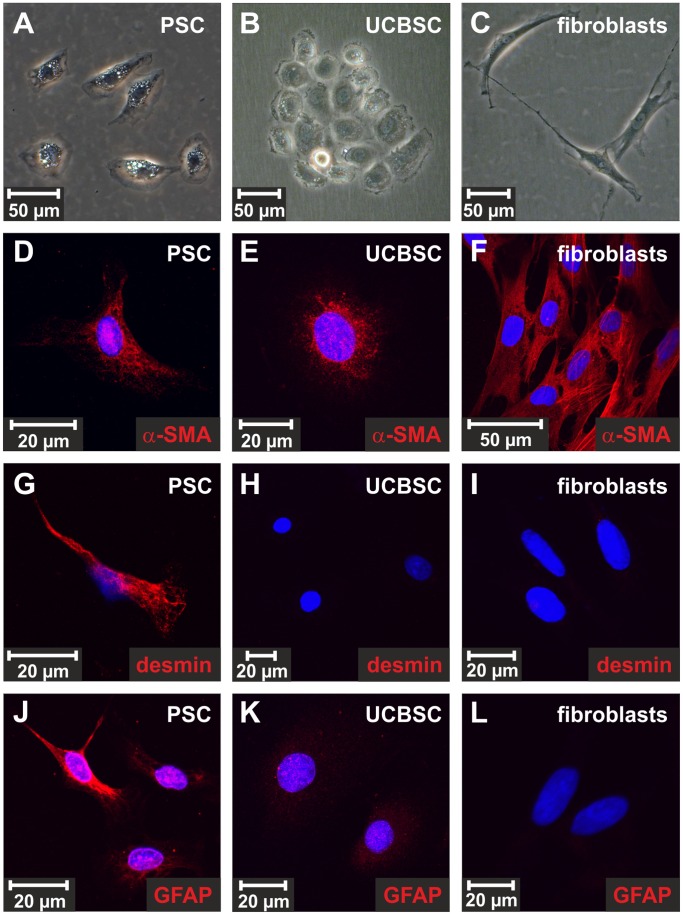
Stellate cell markers in cultured PSC, UCBSC and muscle fibroblasts. (**A,D,G,J**) Freshly isolated PSC cultured for 1 day, (**B,E,H,K**) clonally expanded UCBSC and (**C,F,I,L**) muscle fibroblasts from rats were analyzed by phase contrast light microscopy and antibodies against the stellate cell markers (**D,E,F**) α-SMA, (**G,H,I**) desmin and (**J,K,L**) GFAP (red). The isolated PSC were found to be typical stellate cells with respect to their α-SMA, desmin and GFAP expression. The stellate cell markers α-SMA and GFAP were only weakly expressed by UCBSC clones, whereas desmin remained undetectable at protein level. The cell nuclei were stained by DAPI (blue).

## Materials and Methods

### Cell Sources

PSC, HSC and muscle fibroblasts were isolated from adult male Wistar rats, which were obtained from the local breeding colony. PSC and HSC were enriched by density gradient centrifugation (8% Nycodenz; Nycomed Pharma, Oslo, Norway) after enzymatic digestion of the pancreas or liver essentially as described elsewhere [8], [22]. Isolated PSC were also expanded as single cell clones and maintained in culture for up to 7 months. Stem cells from the umbilical cord blood of unborn Wistar rats (18–20 days *post coitum*) were collected by flushing out the umbilical cord with medium. These umbilical cord blood stem cells (UCBSC) were then clonally expanded and maintained for several months in cell culture. The fibroblasts used in this study derived from small abdominal muscle pieces by outgrowing. Only muscle fibroblasts without nestin and CD146 expression as determined by RT-PCR analysis were used.

**Figure 2:**
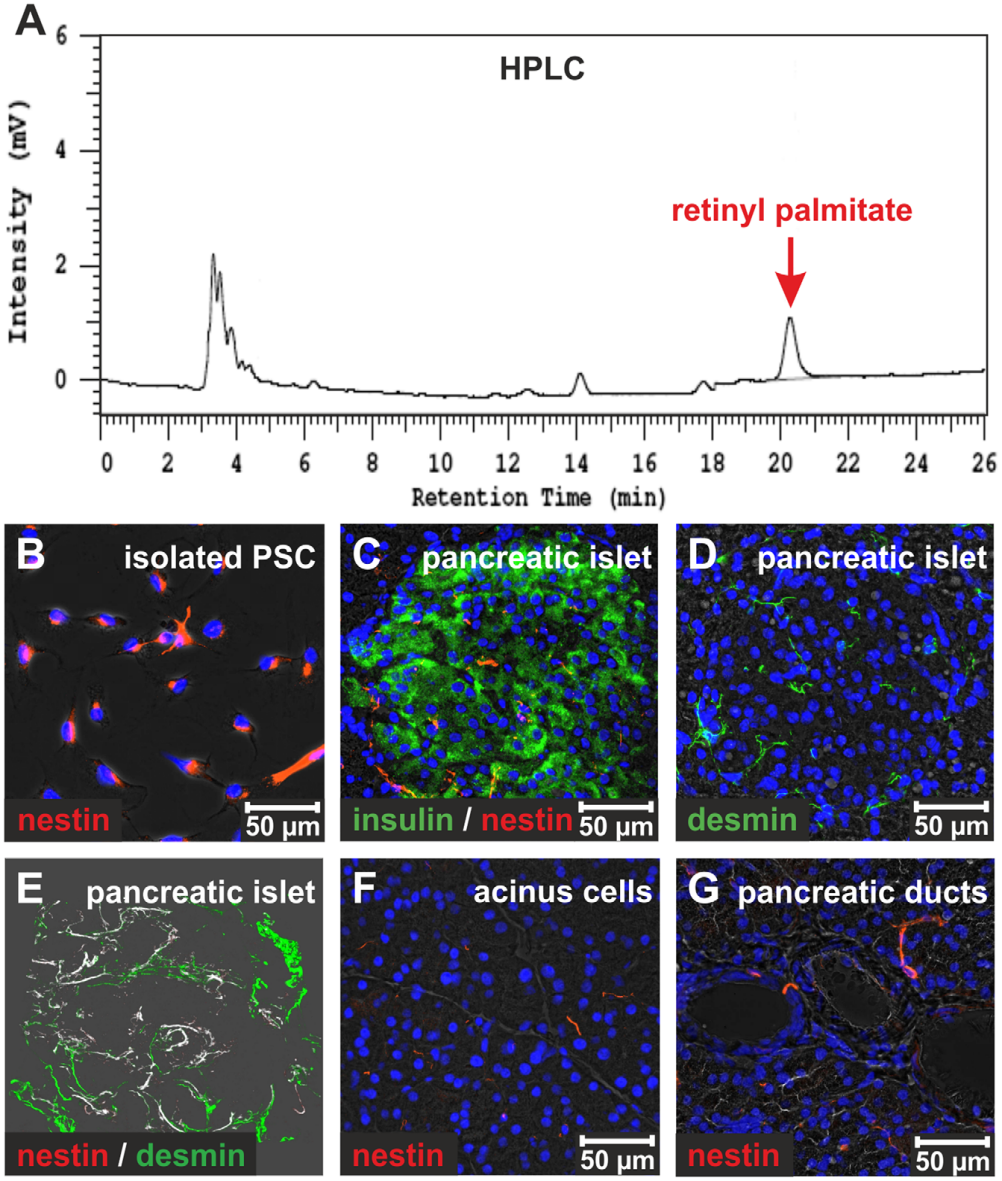
PSC contain retinyl palmitate and express nestin. (**A**) HPLC analysis revealed retinyl palmitate (red arrow) in lysates of freshly isolated rat PSC. (**B**) The isolated PSC were positively labeled with antibodies against nestin (red) at their second day in culture. (**C**) Nestin-expressing cells were mainly found in pancreatic islets of rats (red). Immunofluorescence staining of insulin (green) was used to verify the presence of nestin^+^ cells (red) in pancreatic islets. (**D**) Desmin-expressing cells with branched cell protrusions were observed in pancreatic islets (green). (**E**) The nestin^+^ cells (red) of pancreatic islets were also positive for the stellate cell marker desmin (green) as documented by co-staining of the proteins and 3-dimensional (3D) rendering of serial microscopic pictures made by a confocal laser scanning microscope. The co-localization of nestin and desmin was highlighted in white and indicated the presence of PSC in pancreatic islets. (**F**) Nestin was only detected in few periacinar cells by immunofluorescence staining, (**G**) whereas ductular cells of the rat pancreas were negative for nestin (red). The cell nuclei were marked by DAPI (blue).

### Cell Culture

The UCBSC clones were cultured in Dulbecco’s Modified Eagle Medium Nutrient Mixture F-12 (DMEM-F12; Gibco, Invitrogen, Karlsruhe, Germany) supplemented with 15% fetal calf serum (FCS; Stem Cell Technologies; 06902), 10 ng/ml rat leukemia inhibitory factor (Millipore, Billerica, MA, USA) and 1% antibiotic/antimycotic solution (Gibco; 100x). PSC and muscle fibroblasts were maintained in DMEM supplemented with 10% FCS Gold (PAA Laboratories, Pasching, Austria) and 1% antibiotic/antimycotic solution. To obtain stable clones from single PSC, isolated PSC were cultured for 3 weeks until confluency and then seeded on 96-well plates (plastic ware for the maintenance of embryonic stem cells; Celprogen, San Pedro, CA, USA) at a density of 0.3 cells per well. Immediately after seeding, the separation of PSC into single cells was controlled with a microscope. *In vitro* differentiation of isolated PSC was performed with Iscove’s Modified Dulbecco’s Medium (IMDM) supplemented with 40 ng/ml human HGF (Abcam, Cambridge, UK), 50 ng/ml human FGF_4_ (R&D Systems, Minneapolis, MN, USA), 1% antibiotic/antimycotic solution, 1% linolic acid-albumin and 1% insulin-transferrin-sodium selenite (ITS; Sigma) essentially as described [23], [7]. For direct comparison, PSC were also cultured in IMDM, 10% FCS and 1% antibiotic/antimycotic solution in this experiment (control). The experimental media were exchanged every second to third day.

**Figure 3:**
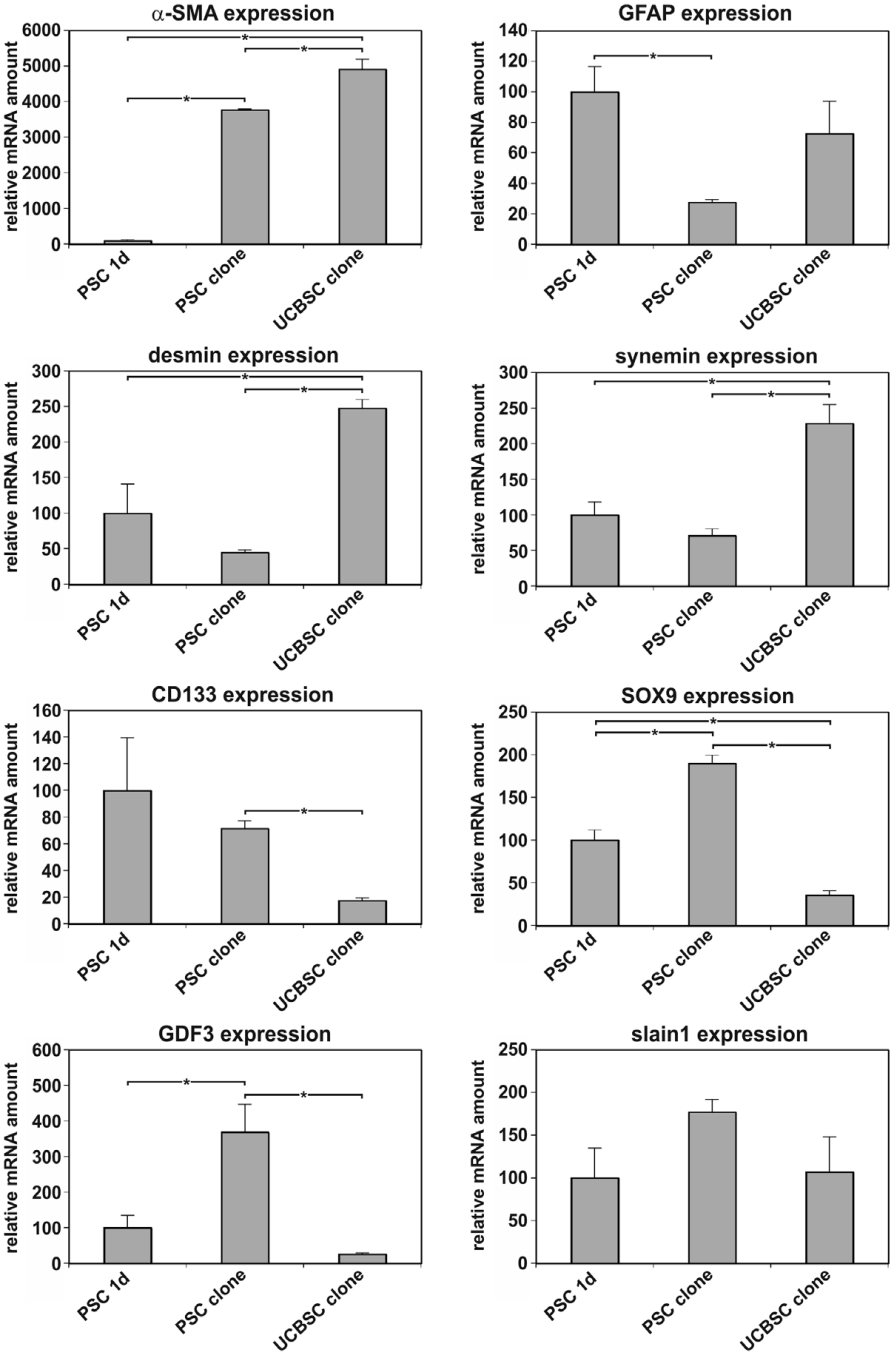
Quantitative mRNA analysis of stellate cell and stem cell-related factors in PSC. The expression of the stellate cell markers α-SMA, GFAP, desmin and synemin as well as the stem/progenitor cell-associated proteins CD133, SOX9, GDF3 and slain1 was investigated by qPCR in one day-cultured PSC, PSC clones and UCBSC clones. PSC expressed comparable or higher levels of stem cell-associated factors than UCBSC clones. Three to five independent primary cultures of PSC were used for qPCR analysis. The PSC clones and the UCBSC clones were measured in triplicates. Significant differences and the standard error of mean (±SEM) are indicated [*P<0.05%].

**Figure 4:**
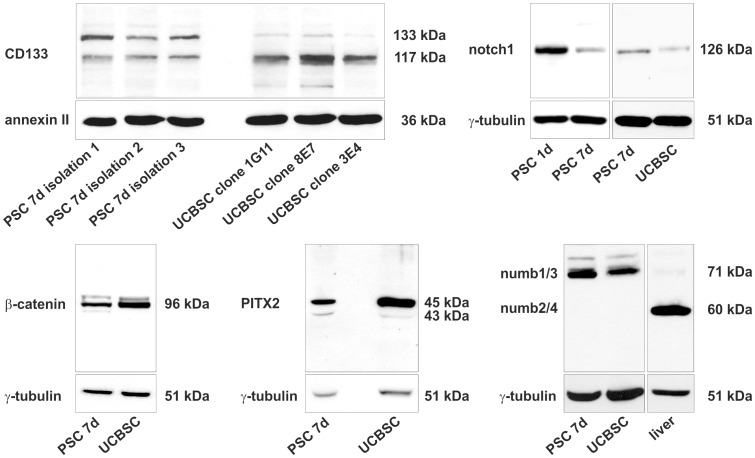
Analysis of stem/progenitor cell-associated factors in PSC by Western blot. The expression of CD133 was analyzed in cell membrane fractions of PSC and UCBSC. PSC of three different cell isolations, which were cultured for 7 days (7d), and UCBSC of three different clones were taken for the immunoblot. To indicate cell membrane protein fractions, annexin II served as a control. The notch1 receptor was detected predominantly in freshly isolated PSC cultured for 1 day (1d) and to a lesser extent also in culture-activated PSC (7d) as well as UCBSC. Nuclear localization of β-catenin in PSC and UCBSC indicated active β-catenin-dependet Wnt signaling as investigated by Western blot of nuclear protein fractions. In line with this, the Wnt target gene *PITX2* was also detected in both cell types. The stem/progenitor cell-associated numb1/3 isoforms were detected in PSC and UCBSC, whereas numb2/4 remained undetectable in both cell types. Numb2/4 isoforms were found in lysates of whole liver.

**Figure 5:**
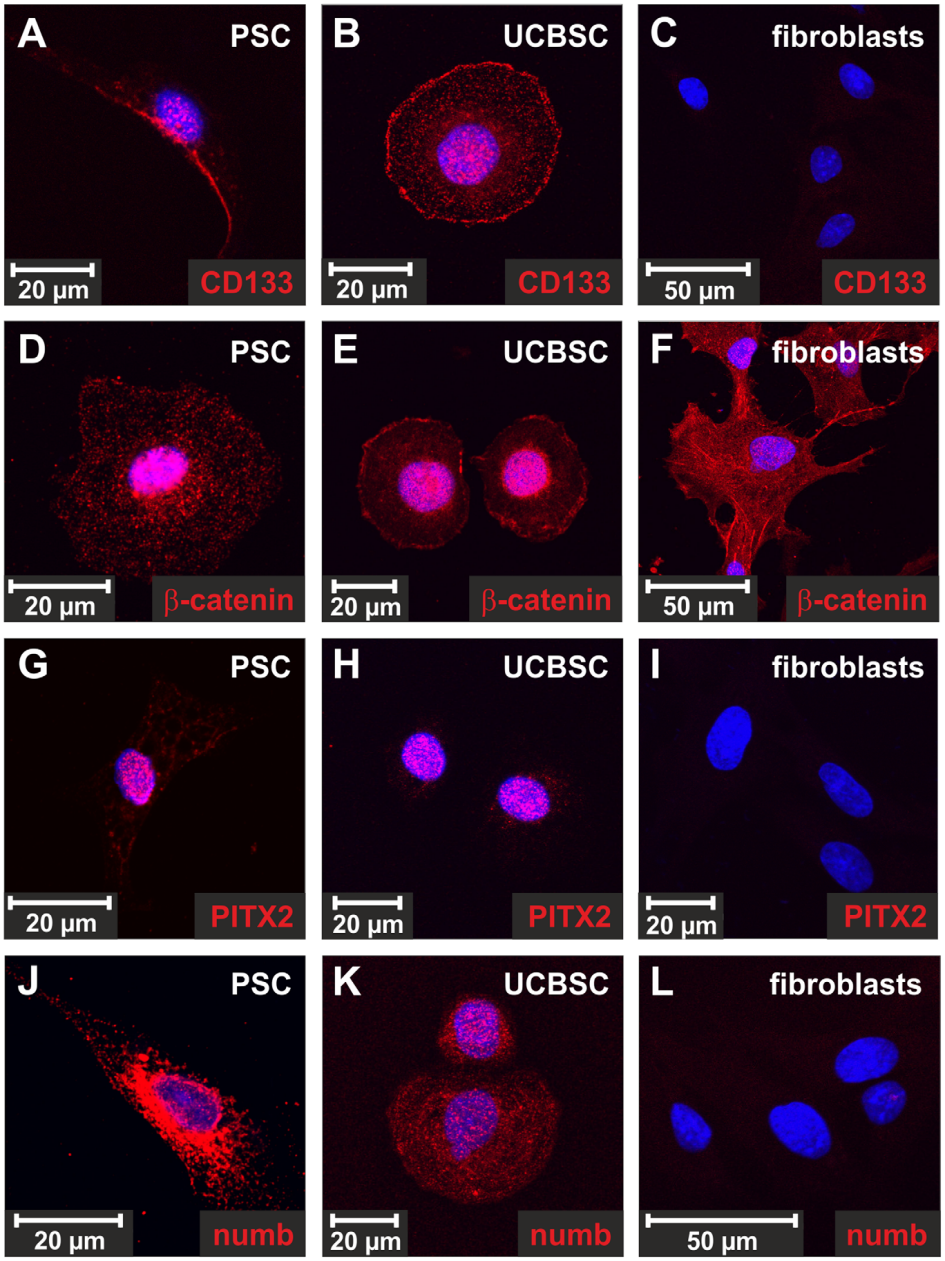
Analysis of stem/progenitor cell-associated factors in PSC by immunofluoresecence. (**A,D,G,J**) Freshly isolated PSC, (**B,E,H,K**) the UCBSC clone 1G11 and (**C,F,I,L**) muscle fibroblasts from rats were analyzed with antibodies against (**A,B,C**) CD133, (**D,E,F**) β-catenin, (**G,H,I**) PITX2 and (**J,K,L**) numb by immunofluorescence staining (red). The stem/progenitor cell-associated proteins CD133, PITX2 and numb were restricted to PSC and UCBSC, whereas β-catenin occurred in all cell types. In (**D**) PSC and (**E**) UCBSC β-catenin was detected in the cell nucleus, indicating active β-catenin-dependent WNT signaling, (**F**) whereas fibroblasts displayed β-catenin mainly in the cell membrane. The cell nuclei were stained by DAPI (blue).

### Western Blot Analysis and Albumin ELISA

Proteins of cell compartments (CNM Compartment Protein Isolation Kit; BioCat, Heidelberg, Germany) and of whole cell lysates were analyzed by Western blot according to standard protocols. Primary antibodies against CD133, numb (Abcam), α-SMA, γ-tubulin (Sigma), glyceraldehyde 3-phosphate dehydrogenase (GAPDH; Millipore) and annexin II (Beckton Dickinson) were used for immunoblots. The release of albumin into the culture medium was measured by a rat-specific albumin enzyme-linked immunosorbent assay (ELISA; Bethyl Laboratories, Montgomery, TX, USA) according to manufacturer’s instructions.

**Figure 6:**
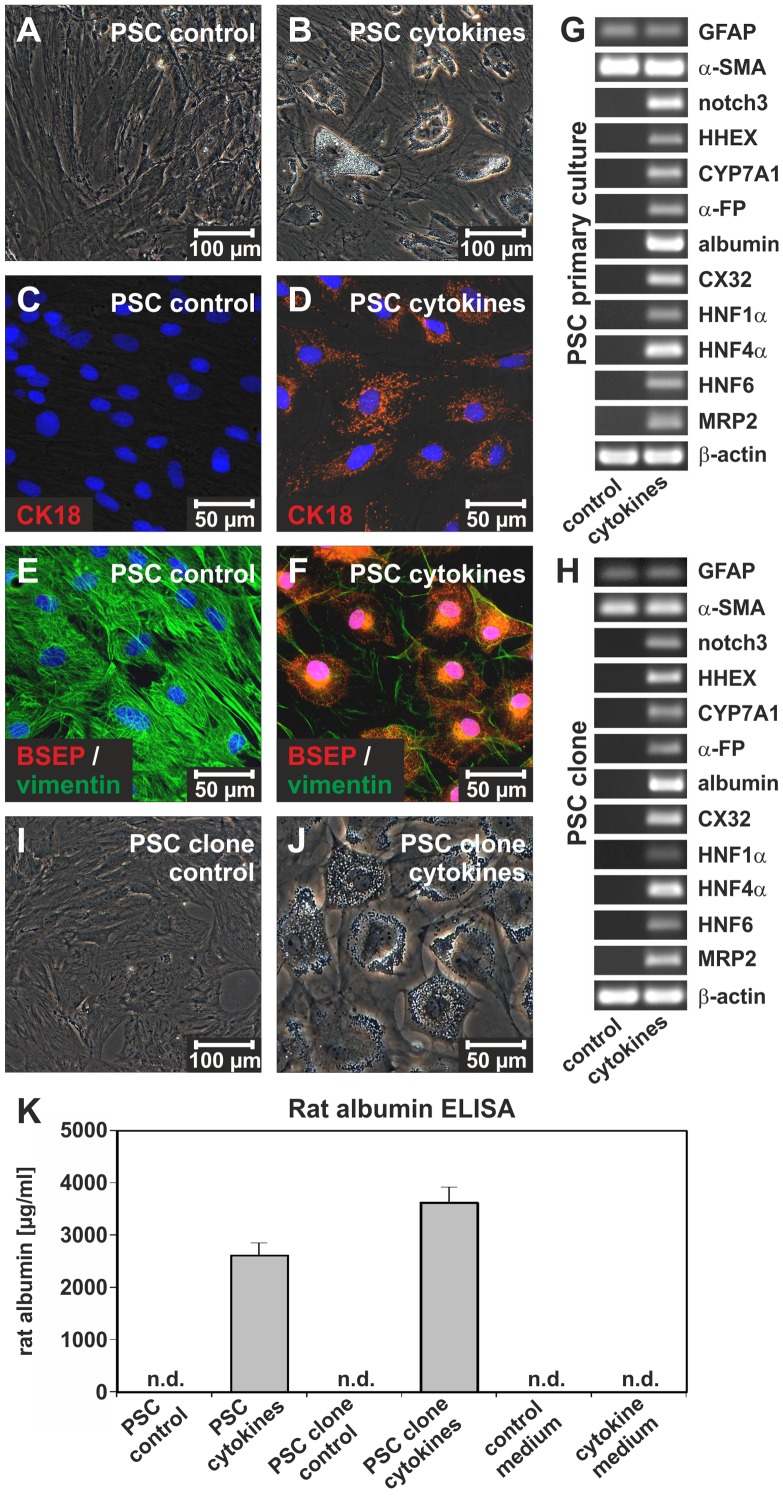
PSC can differentiate into hepatocyte-like cells *in vitro*. PSC were expanded for 7 days in culture and subsequently cultured for additional 14 days in (**A**) IMDM supplemented with 10% FCS (control medium) or (**B**) IMDM containing cytokines (FGF_4_, HGF), linoleic acid-albumin and ITS (hepatocyte differentiation medium). (**B**) Hepatocyte-like cells appeared in primary cultures of PSC during cytokine treatment. (**C**) Under control conditions primary cultured PSC did not express the hepatocyte marker cytokeratin 18 (CK18) as investigated by immunofluorescence (red), but (**D**) CK18 synthesis was induced after treatment with hepatocyte differentiation medium for 14 days. (**E**) The hepatocyte-associated bile salt export pump (BSEP; red) remained undetectable in vimentin-expressing PSC (green) of the control, whereas (**F**) BSEP and vimentin were detectable in PSC treated with hepatocyte differentiation medium. The cell nuclei were marked by DAPI (blue). (**G**) Molecular markers of stellate cells (GFAP, α-SMA) as well as of immature and mature hepatocytes (HHEX, CYP7A1, α-fetoprotein, albumin, CX32, HNF1α, HNF4α, HNF6, MRP2) were analyzed by RT-PCR in the control (IMDM and 10% FCS; left column) and cytokine treated PSC (FGF_4_, HGF, linoleic acid-albumin and ITS; right column) after 14 days of culture. The induction of notch3 and genes typically expressed by liver parenchymal cells indicated cell differentiation. (**H**) This cell differentiation potential was also preserved by the PSC clones, which displayed a comparable expression pattern after cytokine treatment for 7 days (right column). In the control, clonally expanded PSC were cultured in medium without cytokines (IMDM and 10% FCS; left column). (**I**) The PSC clone displayed the morphology of myofibroblast-like cells (control), but (**J**) developed into hepatocyte-like cells after treatment with hepatocyte differentiation medium for 7 days. (**K**) In this experimental setup, the release of albumin was measured by a rat-specific albumin ELISA. High amounts of albumin were secreted by primary cultured PSC and the PSC clone 2F5 after treatment with hepatocyte differentiation medium, whereas rat albumin was not detected (n.d.) under control conditions or in experimental media without cells (n = 3).

### Immunofluorescence and Immunohistochemistry

Isolated cells, cryosections of tissues and cell spheroids were fixed with ice-cold methanol and incubated with antibodies against α-SMA, collagen type I, laminin (Sigma), vimentin (Dako, Glostrup, Denmark), cytokeratin 18 (CK18; Acris, Herford, Germany), CK19 (Progen Biotechnik, Heidelberg, Germany), GFAP (Millipore), insulin (Cell Signaling, Danvers, Ma, USA), β-catenin, CD133, collagen type IV, desmin, epithelial cell adhesion molecule (EP-CAM), epimorphin, numb, thymocyte antigen-1 (THY-1; Abcam), panCK (Biocare Medical, Concord, CA, USA) and nestin (Rat-401; Santa Cruz Biotechnology, Santa Cruz, CA, USA) for immunofluorescence staining. Bile salt export pump (BSEP) was detected by a polyclonal antibody (K12) [24], which was kindly provided by Prof. Dr. Bruno Stieger and Prof. Dr. Peter Meier-Abt. Secondary cyanine dye 3 (Cy3) or fluorescein isothiocyanate (FITC) labeled antibodies (Millipore) and 4′,6-diamidino-2-phenylindole (DAPI; ProLonged Gold; Molecular Probes, Invitrogen) were applied to mark primary antibodies and cell nuclei, respectively. For detection of enhanced green fluorescent protein-expressing (eGFP^+^) cells, the livers were perfused with 4% formalin for 30 minutes, stored in 30% saccharose overnight at 4°C and finally frozen in liquid nitrogen. Since the formalin fixation preserved the fluorescence of eGFP to a certain extent, eGFP^+^ cells were documented in formalin fixed cryosections (10 µm thickness) after excitation with blue light. The eGFP was also detected by immunofluorescence and immunohistochemistry using monoclonal (clone B2, Santa Cruz; clone 3E6, Invitrogen) and polyclonal antibodies (Aves Labs; Tigard, OR, USA) against eGFP followed by labeling with secondary antibodies coupled to Cy3 or FITC. For immunohistochemistry secondary antibodies labeled with alkaline phosphatase (Sigma) and Fast Red tablets that contained Levamisol for blocking endogenous alkaline phosphatase (Roche, Mannheim, Germany) were used. Fast Red stained liver sections were counterstained by diluted hematoxylin solution (Mayer’s; Carl Roth, Karlsruhe, Germany) for 3 minutes. Hematoxylin solution was removed by washing with distilled water and tap water to color the cell nuclei blue. The differentiation of eGFP^+^ PSC was determined by monoclonal antibodies against hepatocyte nuclear factor 4α (HNF4α; Cell Signaling), CK18, CK19 and panCK.

**Figure 7:**
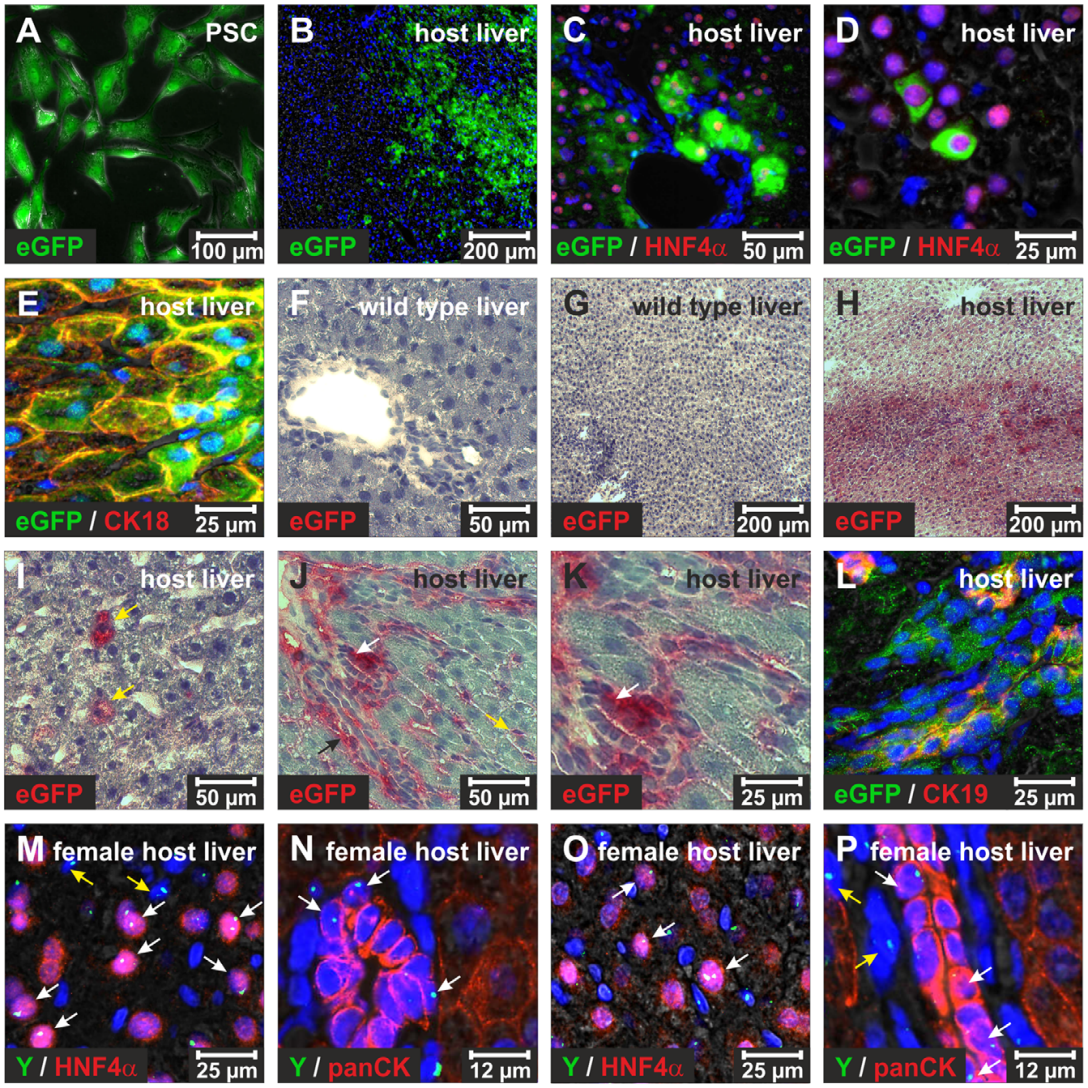
Contribution of transplanted eGFP^+^ PSC to liver regeneration *in vivo*. (**A**) eGFP fluorescence (green) in PSC cultured for 7 days. (**B**) These culture-activated eGFP^+^ PSC were transplanted into wild type rats that underwent PHX in the presence of 2AAF. The eGFP^+^ PSC reached the host liver as determined by immunofluorescence staining of eGFP (green) 14 days after transplantation. (**C, D**) The eGFP^+^ PSC differentiated into hepatocytes in the host liver as determined by co-localization of eGFP fluorescence (green) and nuclear HNF4α immunofluorescence (red). (**E**) Their differentiation into hepatocytes was also confirmed by combined immunofluorescence staining of eGFP (green) and cytokeratin 18 (CK18; red). The fate of transplanted eGFP^+^ PSC was further analyzed by immunohistochemistry through Fast Red staining of eGFP-expressing cells (red). (**F, G**) The liver from wild type Wistar rats displayed no Fast Red staining after incubation with antibodies against eGFP, (**H**) but when eGFP^+^ PSC were transplanted, large areas of the host liver were colored red. This method labeled (**I**) hepatocytes (yellow arrows) and (**J, K**) bile duct cells (white arrows). (**J**) Other cell types in close proximity to bile ducts (black arrow) and liver sinusoids (yellow arrow) showed also eGFP expression as indicated by Fast Red staining. (**L**) The presence of eGFP-expressing cells in bile ducts of the host liver was further confirmed by combined immunofluorescence of eGFP (green) and CK19 (red). (**M**) Immunofluorescence staining of HNF4α (red) and FISH of chromosome Y (green) was used to identify hepatocytes in female liver tissue that derived from transplanted male PSC 14 days after PHX in the presence of 2AAF (white arrows). Also non-parenchymal cells with chromosome Y were detected (yellow arrows). (**N**) Combined FISH of chromosome Y (green) and immunofluorescence staining of panCK (red) revealed that the transplanted PSC had differentiated into duct-forming cholangiocytes. (**O, P**) Clonally expanded PSC of males were also transplanted into wild type female rats that underwent PHX in the presence of 2AAF. FISH of chromosome Y (green) and immunofluorescence staining of (**O**) HNF4α (red) or (**P**) panCK (red) indicated that single cell clones of PSC differentiated into hepatocytes and cholangiocytes (white arrows) as investigated after 14 days of regeneration. (**P**) Also PSC-derived cells that did not express cytokeratins were found close to bile ducts (yellow arrows). The cell nuclei were marked by DAPI (blue).

### Flow Cytometry

Freshly isolated PSC and HSC from male Wistar rats (500–600 g bodyweight) were analyzed by the flow cytometer MoFlo XDP (Beckman Coulter, Krefeld, Germany). Stellate cells from pancreas and liver that fulfilled similar morphological properties as determined by forward and side scatter were excited by an UV laser (350 nm) to measure retinoid fluorescence at 485 nm.

**Figure 8:**
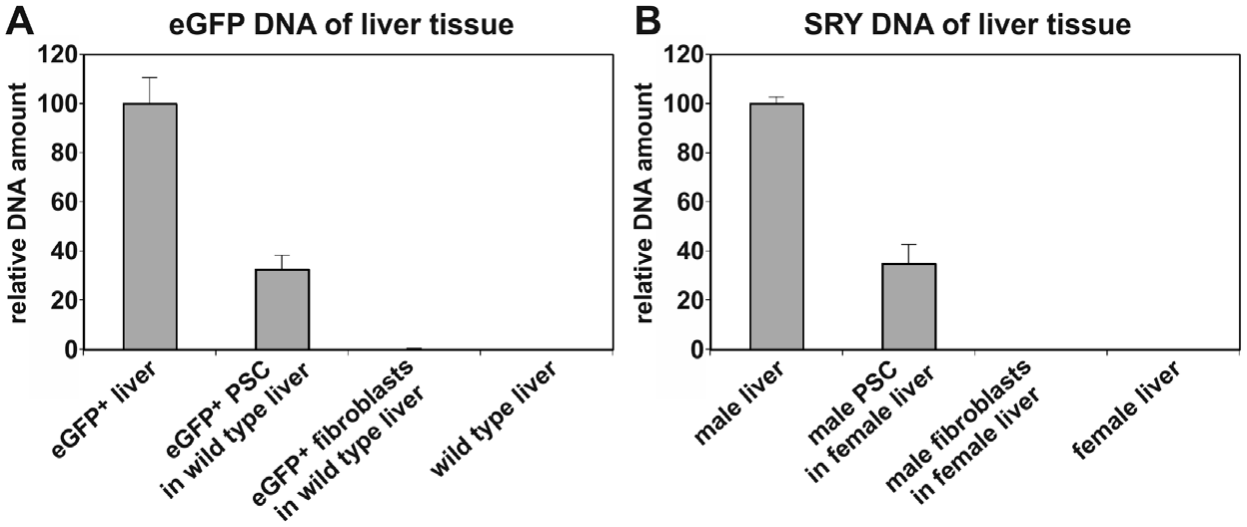
Estimation of PSC-derived cells in the wild type host liver. (**A**) In order to quantify the contribution of transplanted eGFP^+^ PSC to liver regeneration, qPCR of the eGFP DNA was performed 14 days after cell transplantation. Livers from eGFP-expressing and wild type rats served as positive and negative controls for the eGFP gene, respectively. Ten samples of different animals were measured to assess the eGFP expression of liver after PSC transplantation. (**B**) The engraftment of stellate cells was further verified through qPCR of male-specific SRY DNA after gender-mismatched transplantation of male PSC into female recipient rats (n = 4). In contrast to PSC, muscle fibroblasts were unable to survive in the host liver (n = 4).

### Polymerase Chain Reaction

The first strand cDNA was made from 1 µg total RNA per 20 µl reaction volume (RevertAid H Minus First Strand cDNA Synthesis Kit; Fermentas, St. Leon-Rot, Germany) for reverse transcriptase polymerase chain reaction (RT-PCR). The PCR was performed according to standard protocols using the 2xPCR Master Mix (Fermentas), 0.8 µmol/l primers (Table S1) and 1 or 5 µl template cDNA per 25 µl reaction volume. A maximum of 35 cycles was used for amplification of PCR products. Quantitative real-time PCR (qPCR) was performed using the SensiMix SYBR No-ROX Kit (Bioline, Luckenwalde, Germany) according to manufacturer’s recommendations. DNA was purified from rat liver using the DNeasy Blood & Tissue Kit (Qiagen, Hilden, Germany). For each sample 12.5 ng cDNA or 100 ng DNA and 0.6 µmol/l primers (Table S2) were used. After denaturation at 95°C, the annealing was performed at 58°C or 60°C and the elongation was conducted at 72°C using a TOptical cycler (Biometra, Göttingen, Germany). All samples were analyzed in triplicates and hypoxanthine-guanine phosphoribosyltransferase 1 (HPRT1; mRNA of liver tissue), ribosomal protein S6 (RPS6; mRNA of isolated cells) or β-actin (DNA of liver tissue) were used as reference genes for the normalization of the results obtained by the 2(−ΔΔCt) method.

### High-performance Liquid Chromatography Analysis

For the analysis of retinoids by high-performance liquid chromatography (HPLC), the stellate cells were suspended in phosphate buffered saline, sonicated on ice for 20 seconds and fat soluble vitamins were extracted by hexane/dichlormethane (5/1) containing 0.01% butylated hydroxytoluene. For the analysis of retinol and retinyl esters a HPLC system with a LaChrom L-7100 gradient pump and LaChrom UV/Vis detector L-7420 (Merck–Hitachi) was used. The analytic column was a Suplex pKb-100 (25 cm×2.1 mm, Supelco) equipped with a guard column to prevent particulate matter. The solvent system consisted of a linear methanol/acetonitrile/2-propanol (54/44/2) and methanol/acetonitrile/H_2_O/2-propanol (46/37/15/2) gradient and was used at a flow rate of 1 ml/min.

### Fluorescence *in situ* Hybridization

Fluorescence *in situ* hybridization (FISH) of the chromosome Y was performed on cryosections of livers after fixation with 75% methanol and 25% acetic acid at −20°C for 15 minutes and subsequent digestion of the tissue with 0.05 mg/ml pepsin (Roche) in 0.01 N chloric acid at 37°C for 1–2 minutes. The fluorescent probe of chromosome Y (MetaSystems, Mannheim, Germany) dissolved in a hybridization solution (H7782; Sigma) was added to the sections, which were subsequently covered with a coverslip, sealed with rubber cement, heated at 75°C for 3 minutes and finally incubated at 37°C overnight. After removal of the coverslip the sections were washed in 0.4-fold saline sodium citrate (SSC) at 72°C for 2 minutes, in 2-fold SSC with 0.05% Tween 20 at 20°C for 1 minute and finally rinsed in distilled water. Proliferating PSC of males and females were arrested in metaphase through addition of 0.08 µg/ml colchicine (Sigma) for 6 hours. The cells were washed twice with preheated 0.5% potassium chloride solution, incubated for 20 minutes at 37°C and finally fixed with 75% methanol and 25% acetic acid at −20°C for 20 minutes before FISH of sex chromosomes was performed.

### Transplantation of eGFP^+^ Cells

The animal procedures were approved by the local committee for animal protection (Landesamt für Natur-, Umwelt- und Verbraucherschutz, Nordrhein-Westfalen, Germany; Permit Number: 9.93.2.10.34.07.163) and all animals received care according to the German animal welfare act. All surgery was performed under ketamine/xylazine anesthesia and carprofen application. Wistar rats (Wistar-TgN(CAG-GFP)184Ys) expressing eGFP under the control of a chicken β-actin promoter were obtained from the Rat Resource & Research Center (University of Missouri, Columbia, MO, USA) and used as a source for eGFP^+^ cells. Pellets containing 2-acetylaminofluorene (2AAF; 70 mg 2AAF 14 day release; Innovative Research of America, Sarasota, FL, USA) were implanted under the skin of the neck of wild type Wistar rats (approximately 250 g body weight) 7 days before partial hepatectomy (PHX; surgical removal of the two largest liver lobes or 70–80% of the liver mass) was performed essentially as described [25], [26]. Cells of single PSC clones, PSC primary cultures or muscle fibroblasts from eGFP^+^ Wistar rats were transplanted into wild type Wistar rats via tail vein injections immediately after PHX. About 0.4×10^6^ to 4×10^6^ PSC, 5×10^6^ clonally expanded PSC or 7–10×10^6^ muscle fibroblasts were transplanted into the recipient animal. Prior to transplantation the cells were treated with medium that contained rat serum and 10 ng/ml interleukin-6 (R&D Systems) for 1 day.

### Statistics

The data were analyzed using the Students t-test and considered significant at p<0.05. The results of at least 3 independent experiments were expressed as means (± standard error of mean; SEM).

## Results

### Identification of PSC

Freshly isolated PSC from rats possess only few lipid droplets (Fig. 1A) and contain low levels of retinyl palmitate (0.8±0.4 µmol/mg protein; n = 7) as determined by HPLC (Fig. 2A), whereas the retinyl palmitate content of freshly isolated HSC is 80 times higher [7]. As shown by flow cytometry using the retinoid autofluorescence (excited at 350 nm), freshly isolated PSC showed a low retinoid content compared to freshly isolated HSC (Fig. S1). Stellate cells characteristically express GFAP, desmin, synemin and vimentin, which are also detectable in isolated PSC in addition to the myofibroblast marker α-SMA (Fig. 1D,G,J; Fig. S2). Like PSC, clonally expanded stem cells of the umbilical cord blood (UCBSC) show low α-SMA and GFAP protein amounts (Fig. 1E,K). Albeit the mRNA of desmin was detectable by RT-PCR, the UCBSC clones were negative for desmin at protein level (Fig. 1H). For comparison, cultured muscle fibroblasts strongly expressed α-SMA, whereas desmin and GFAP were undetectable (Fig. 1F,I,L). Nestin is another filamentous protein synthesized by activated stellate cells from liver and pancreas. Isolated PSC could be marked by antibodies against nestin at their second day of culture, because nestin was immediately up-regulated during activation of PSC in culture (Fig. 2B; Fig. S2). According to immunofluorescence stainings of these stellate cell markers, primary cultures of rat PSC were found to be highly uniform (uniformity 97%) and pancreatic duct-associated stem/progenitor cells were absent as indicated by the lack of cytokeratins (panCK; Fig. S2; Table S3). In order to identify nestin-expressing PSC in the rat pancreas, immunofluorescence staining of nestin was performed. The nestin-positive cells that co-expressed the stellate cell marker desmin were mainly found in pancreatic islets (Fig. 2C–E), but also some periacinar cells and very few cells at the pancreatic ducts expressed nestin (Fig. 2F,G).

### Stem/progenitor Cell Markers in PSC

One characteristic feature of stem/progenitor cells is the expression of genes associated with developmental processes. For this reason freshly isolated PSC were analyzed by RT-PCR and the expression of the stem/progenitor cell markers breast cancer resistance protein 1 (BCRP1), bone morphogenetic protein-binding endothelial regulator (BMPER), CD133, c-kit, CXC chemokine receptor 4 (CXCR4), growth/differentiation factor 3 (GDF3), nestin, notch1, paired-like homeodomain transcription factor 2c (PITX2c), runt-related transcription factor 1 (RUNX1), stem cell growth factor (SCGF), slain1 and SRY-box 9 (SOX9) was found (Fig. S3). The mRNAs of proteins that are frequently used to characterize mesenchymal stem cells (CD29, CD73, and CD146) were also detected in freshly isolated PSC. A similar expression profile was found in UCBSC clones, but not in muscle fibroblasts of rats (Fig. S3). The analysis of the mRNA amount in freshly isolated PSC by quantitative PCR (qPCR) revealed that the stellate cell markers α-SMA, GFAP and desmin were also highly expressed by PSC and UCBSC. In freshly isolated PSC the stem/progenitor cell-associated proteins nestin, CD133, SOX9, GDF3 and slain1 reached comparable or even higher mRNA levels than in UCBSC (Fig. 3). The expression of CD133, notch1, PITX2 and numb by PSC and UCBSC clones was verified by Western blot analysis and immunofluorescence staining (Fig. 4; Fig. 5A,B,G,H,J,K). The cell membrane protein fractions of activated PSC displayed two CD133 protein bands at approximately 117 and 123 kDa, whereas the 117 kDa protein band predominated in UCBSC clones (Fig. 4). PSC showed two CD133 transcript variants as determined by sequencing of PCR products (Fig. S4), which probably explains the presence of two protein bands in Western blot analysis (Fig. 4). These CD133 transcript variants appeared during activation of PSC in culture, but their relevance is still unknown. Transcript variants were also observed for numb, which is a modulator of notch signaling. Large transcript variants of numb (numb isoforms 1/3; approximately 71 kDa) were expressed by PSC and UCBSC clones, whereas the short numb isoforms 2/4 (about 60 kDa), which are known to be induced during cell differentiation [27], were absent (Fig. 4). The presence of notch1 and numb suggests that PSC possess jagged/delta-like-dependent notch signaling pathways. Western blot and RT-PCR revealed the expression of notch1 mainly in freshly isolated PSC (Fig. 4; Fig. S3). Nuclear β-catenin and its target gene *PITX2* indicated active β-catenin-dependent WNT signaling in freshly isolated PSC and UCBSC clones (Fig. 5D,E,G,H). As shown in embryonic stem cells, this WNT signaling pathway is required for the maintenance of stemness [28]. In contrast to PSC and UCBSC, in muscle fibroblasts β-catenin is predominantly localized in the plasma membrane and not in the nucleus (Fig. 5F). Numb was found primarily in the cytosol of PSC and UCBSC (Fig. 5J,K), whereas muscle fibroblasts were negative for CD133, PITX2 and numb (Fig. 5C,I,L).

### Clonal Growth of PSC

Another characteristic feature of stem cells is their ability to grow as cell clones. In order to test for this, single PSC were cultured in 96-well plates, expanded in larger culture dishes and finally characterized. PSC displayed a high cloning efficiency and their clones survived for more than 7 months in culture. All PSC clones initially displayed the morphology of myofibroblast-like cells, but developed also into small round cells later during culture. Confluent PSC clones frequently formed cell spheroids as observed in primary cultures of PSC (Fig. S5). The PSC clones expressed the stellate cell markers α-SMA, GFAP and desmin along with the stem/progenitor cell makers nestin, CD133, SOX9, GDF3 and slain1 as determined by qPCR (Fig. 3). Like freshly isolated PSC, clonally expanded PSC were negative for panCK and EP-CAM, but expressed mesodermal proteins such as vimentin (not shown).

### Differentiation of PSC *in vitro*

The ability of PSC to undergo developmental processes as a typical hallmark of stem/progenitor cells was investigated *in vitro*. In order to induce cell differentiation, isolated PSC were expanded for 7 days in culture and then treated with the cytokines FGF_4_ and HGF as well as the supplements linoleic acid-albumin and ITS (hepatocyte differentiation medium) for another 14 days. Treatment with this cell differentiation medium changed the morphology of PSC when compared to culture in control medium (Fig. 6A,B). Immunofluorescence of the hepatocyte markers CK18 and BSEP suggested that PSC developed into hepatocyte-like cells after cytokine treatment (Fig. 6C-F), but the mesodermal protein vimentin was still detectable in PSC-derived cells with hepatocyte markers (Fig. 6F). The cytokines decreased stem/progenitor cell factors (e.g. GDF3 and slain1; not shown), but induced notch3 and the hepatocyte markers hematopoietically-expressed homeobox protein (HHEX), cytochrome P450 7A1 (CYP7A1), α-fetoprotein (α-FP), albumin, connexin 32 (CX32), hepatocyte nuclear factor 1α (HNF1α), HNF4α, HNF6 and multidrug resistance protein 2 (MRP2) as investigated by RT-PCR (Fig. 6G). A similar response to cytokine treatment was induced in clonally expanded PSC (Fig. 6H), indicating that clonally expanded PSC preserved their differentiation potential. In contrast to primary cultured PSC, clear changes in cell morphology in the majority of clonally expanded PSC were already observed 7 days after treatment with hepatocyte differentiation medium (Fig. 6I,J). PSC-derived hepatocyte-like cells were able to synthesize albumin as determined by a rat albumin-specific ELISA, suggesting that typical hepatocyte functions became apparent (Fig. 6K).

Prolonged culture of PSC and PSC clones in the presence of FCS was associated with the formation of cell spheroids. In order to characterize these structures, which are frequently observed in stem cell cultures [4], [29], cryosections of cell spheroids were analyzed. There was a strong immunofluorescence of the basal lamina proteins laminin and collagen type IV. A large proportion of the cells in the spheroids expressed THY-1, nestin, desmin, vimentin and epimorphin (Fig. S5).

### Differentiation of Transplanted PSC *in vivo*

Stem/progenitor cells are transplantable, exhibit long-lasting survival in host tissues and can contribute to tissue regeneration through differentiation. These features were also found in pancreatic stellate cells. PSC were isolated from eGFP^+^ male rats, expanded for 7 days in culture (Fig. 7A) and then transplanted into wild type rats via tail vein injection immediately after PHX. The animals were treated with 2AAF 7 days before PHX to prevent the proliferation of hepatocytes and to facilitate liver regeneration through stem/progenitor cell compartments. The transplanted eGFP^+^ PSC reached the host liver and reconstituted large areas of the injured organ 14 days after their transplantation mainly around portal tracts as investigated by eGFP immunofluorescence (Fig. 7B). The developmental fate of transplanted eGFP^+^ PSC was analyzed in the host liver by eGFP fluorescence and immunofluorescence staining of HNF4α. eGFP^+^ cells that displayed nuclear HNF4α and the morphology of hepatocytes were detected in the wild type host liver (Fig. 7C,D). The differentiation of eGFP^+^ PSC into hepatocytes was further confirmed by combined immunofluorescence staining of eGFP and CK18 (Fig. 7E). The presence of transplanted eGFP^+^ PSC in the host liver was also analyzed by immunohistochemistry using the dye Fast Red. After eGFP antibody labeling, this dye colors PSC-derived cells red and allows their detection independent of fluorescence staining methods. Through bright field microscopy no dye deposition was found in the normal wild type rat liver (Fig. 7F,G), but extensive Fast Red staining was observed in the host liver 14 days after transplantation of eGFP^+^ PSC (Fig. 7H). Not only hepatocytes, but also bile duct cells and other non-parenchymal cells close to ductular structures and in liver sinusoids were eGFP positive, suggestive that they originated from eGFP^+^ PSC (Fig. 7J,K). The identity of eGFP-expressing bile duct cells was confirmed by combined immunofluorescence of eGFP and the cholangiocyte marker CK19 (Fig. 7L). These findings were also supported by FISH of chromosome Y from male PSC (Fig. 7M,N), which were transplanted into females. Prior to this analysis, the chromosome Y probe was validated in metaphase spreads of male and female PSC (Fig. S6). Transplanted male PSC had differentiated into hepatocytes as suggested by their HNF4α expression (Fig. 7M). Cells with chromosome Y were also detected in duct-forming cells with high panCK expression, indicating that transplanted PSC also developed into cholangiocytes (Fig. 7N). In order to rule out a contribution of other cell types to the observed participation of transplanted PSC to liver regeneration, clonally expanded male PSC were transplanted using the above mentioned liver regeneration model. Transplanted PSC clones also differentiated into HNF4α^+^ hepatocytes and panCK^+^ cholangiocytes as investigated by FISH analysis of chromosome Y (Fig. 7O,P). Within the parenchymal tissue and close to bile ducts other PSC-derived cells without HNF4α or cytokeratin expression were also observed (Fig. 7M,P).

In order to estimate the contribution of transplanted eGFP^+^ PSC to liver regeneration the eGFP DNA was quantified by qPCR in the wild type host liver. Compared to the mean eGFP DNA level found in livers from transgenic eGFP^+^ rats, the amount of eGFP DNA in the host liver reached 33±6% 14 days after transplantation of eGFP^+^ PSC, suggesting high abundance of PSC-derived cells in the recipient liver (Fig. 8A). The eGFP expression remained detectable for at least 28 days as determined by qPCR (not shown), indicating long-lasting survival of transplanted PSC. On the other hand, eGFP^+^ muscle fibroblasts did not integrate into host liver after transplantation (Fig. 8A). Similar results were obtained through determination of male specific SRY DNA located on the chromosome Y after gender-mismatched transplantation of male PSC into female rats that underwent PHX and received 2AAF. Quantitative PCR of genomic SRY DNA revealed that about 35±8% of the liver cells carried the Y chromosome in the recipient female liver 14 days after transplantation of male PSC (Fig. 8B).

## Discussion

There is ongoing discussion about the identity of stem cells in the pancreas. Here we show that essential properties of stem cells are fulfilled by stellate cells of the pancreas. Isolated PSC were found to be typical stellate cells with respect to their low but detectable retinyl palmitate content and expression of filamentous proteins (GFAP, desmin, synemin, vimentin, nestin). They express multiple stem/progenitor cell markers such as CD133 and nestin as well as factors involved in developmental processes (e.g. GDF3 and PITX2), which are present also in established stem cells such as UCBSC. Moreover, PSC display β-catenin-dependent WNT and notch signaling pathways, which are required for stem cell maintenance and development. The nuclear localization of β-catenin and the expression of WNT target genes (e.g. *PITX2*) in freshly isolated PSC indicates an active β-catenin/WNT signaling pathway. The expression of the notch receptors 1 and 3 further suggests notch signaling in PSC. Notch1 is essential for the maintenance of stem cells [30], whereas notch3 is involved in cell development [31].

Another feature of stem/progenitor cells is their ability to undergo developmental processes. Indeed, the typical expression profile of differentiated cells such as hepatocytes can be induced in primary cultures of PSC and PSC clones by FGF_4_ and HGF. This differentiation process was accompanied by a switch from notch1 to notch3 expression, suggesting that PSC were prepared for cell differentiation. However, although hepatocyte markers were detectable and albumin was released into the culture medium, mesodermal proteins such as vimentin were still present, indicating that PSC-derived hepatocyte-like cells remained immature *in vitro*. On the other hand, this intermediate state of hepatocyte-like cells demonstrates the transition of stellate cells from the mesoderm to the endoderm.

Transplantability and contribution to tissue regeneration are further characteristics of stem/progenitor cells. These features were tested using eGFP^+^ PSC, which were transplanted into wild type rats via tail vein injections. Prior to this, the host animals underwent PHX in the presence of 2AAF, which is an established model of stem/progenitor cell-based liver regeneration with inhibition of hepatocyte proliferation in rats [26], [32]. The transplanted eGFP^+^ PSC reached the injured liver presumably owing to their CXCR4 expression (Fig. S3). Especially the injured liver releases high amounts of stromal cell-derived factor 1 [33], which may attract transplanted PSC as a ligand of CXCR4. In the host liver, transplanted eGFP^+^ PSC survived over long time periods (i.e. at least 28 days) and differentiated into HNF4α^+^ hepatocytes or panCK^+^ cholangiocytes that formed bile ducts. Similar results were obtained by transplanted PSC clones, suggesting that stellate cells contributed to liver repair through differentiation. Other cell types of the host liver associated with bile ducts and liver sinusoids were also found to derive from transplanted PSC. The absence of cytokeratins suggests that these cells belong to the mesenchymal tissue. As shown by qPCR analysis of eGFP and SRY DNA, transplanted PSC were able to reconstitute large areas of the injured host liver, indicating highly effective contribution of stellate cells to liver regeneration in the 2AAF/PHX model.

Taken together, these results demonstrate that PSC can contribute to liver repair through differentiation. Mesenchymal stem cells (MSC) from bone marrow are known to express nestin and can differentiate into hepatocytes [34], [35]. These MSC are characterized by the molecular markers CD29, CD73 and CD146 [36], [37], which were also found in PSC. Further studies are required to determine functional similarities of PSC and MSC, since MSC are primarily defined by their differentiation potential rather than by molecular markers. The similar expression pattern of PSC and established stem cells such as UCBSC as well as the clonal growth of PSC as demonstrated in the present study suggests that stellate cells resemble stem cells rather than progenitor cells. This view is also supported by the potential of PSC to self-renew as indicated by their clonal growth with simultaneous maintenance of their developmental potential.

PSC seem to be distinct from CK^+^ progenitor cells associated with pancreatic ducts [38], because freshly isolated and clonally expanded PSC were negative for cytokeratins. As demonstrated in the present study, nestin^+^/desmin^+^ PSC were detectable in pancreatic islets, which are normally devoid of CK^+^ cells, but are known to contain stem cells [4]. Fate mapping experiments that follow-up SOX9-expressing cells indicated the occurrence of this transcription factor in progenitor cells of pancreas, liver and intestine. Using this method, SOX9 expression was initially mainly observed in CK^+^ cells of pancreatic ducts and centroacinar cells [38]. However, the expression of SOX9 was reported for cultured stellate cells from rat liver [39]. In addition, isolated PSC and PSC clones (Fig. 3) as well as HSC (not shown) express SOX9 mRNA, but neither PSC nor HSC were labeled in the study of Furuyama and colleagues [38]. This study relied on α-SMA to identify stellate cells. Since α-SMA is not restricted to PSC or HSC, additional experiments with a combination of more specific markers for activated stellate cells (e.g. nestin, desmin) are required to resolve this discrepancy.

In the present study, we demonstrate that PSC fulfill essential characteristics of stem cells such as (I) an expression profile characteristic for stem cells, (II) clonal cell growth, (III) transplantability with long-lasting survival and (IV) contribution to regenerative processes. PSC can directly contribute to liver regeneration through differentiation into hepatocytes and duct-forming cholangiocytes. This may point to a functional relationship between stellate cells and hitherto known CK^+^ liver progenitor cells. Keeping in mind that stellate cells occur in many organs, the observed participation of PSC in regenerative processes across tissue boundaries could represent a general repair mechanism of the body.

## Supporting Information

Figure S1 **Flow cytometry analysis of retinoid fluorescence in freshly isolated PSC and HSC.** (**A**) PSC and (**B**) HSC of Wistar rats were analyzed by forward and side scatter with identical settings. Stellate cells with similar morphological properties were excited at 350 nm to measure retinoid fluorescence at 485 nm. In contrast to HSC (gate R8), PSC generally contained low retinoid amounts. The analysis was performed with the flow cytometer MoFlo XDP (Beckman Coulter).
(TIF)

Figure S2 **Characterization of PSC by immunofluorescence staining to assess the uniformity of primary cultures.** The PSC were analyzed by immunofluorescence at the second day of culture using antibodies against the stellate cell markers (**A**) α-SMA, (**B**) GFAP, (**C**) synemin, (**D**) vimentin and (**E**) nestin. These proteins were detected in the vast majority of cells, indicating highly uniform PSC preparations. (**F**) Antibodies against cytokeratins were used to exclude the pancreatic duct-associated stem/progenitor cells. The number of positive PSC obtained by this method is presented in Table S3.
(TIF)

Figure S3 **Expression of stem/progenitor cell markers in early primary cultures of PSC.** RT-PCR analysis of stem/progenitor cell markers in one-day-cultured PSC, clonally expanded UCBSC and muscle fibroblasts from rats. Freshly isolated PSC and the UCBSC clone 1G11 displayed a similar expression pattern, which was distinct from differentiated cells such as muscle fibroblasts.
(TIF)

Figure S4 **Sequence analysis of CD133 transcript variants in stellate cells at the mRNA and protein level.** (**A**) During culture of PSC two transcript variants of CD133 became detectable by RT-PCR. (**B**) The shorter transcript variant of CD133 lacks 93 nucleotides (highlighted in red) as investigated by sequencing of the mRNA products obtained by application of the 3′-terminal primers for CD133. This mRNA sequence corresponded to the transcript variant 2 of rat CD133 (gene bank accession number: NM_001110137). (**C**) The resulting difference of both CD133 transcript variants was highlighted (red) in the complete CD133 amino acid sequence and may explain the appearance of two CD133 protein bands in PSC cultured for 7 days (Fig. 4).
(TIF)

Figure S5 **PSC and PSC clones form cell spheroids** ***in vitro*** **.** (**A**) PSC in primary culture formed cell spheroids in the presence of FCS. Cryosections of PSC spheroids were analyzed with antibodies against (**B**) laminin, (**C**) collagen type IV, (**D**) THY-1, (**E**) nestin, (**F**) desmin, (**G**) vimentin and (**H**) epimorphin (red) after 21 days of culture. The cell nuclei were marked by DAPI (blue). (**I**) Also clonally expanded PSC (clone 2F5) developed cell spheroids after 14 days of FCS treatment, indicating that spheroid formation is an intrinsic property of stellate cells.
(TIF)

Figure S6 **Specificity of gender specific DNA probes.** FISH of chromosome Y and X was performed on metaphase spreads of isolated PSC from male and female rats. (**A**) One chromosome was labeled by chromosome Y probes in male PSC (green; arrow). (**B**) The chromosome X probe marked a single chromosome in male PSC (red; arrow). (**C**) Female PSC were without clear labeling after application of the chromosome Y probe (green), (**D**) but displayed two chromosomes, which were bound by the chromosome X probe (red; arrows).
(TIF)

Table S1 **Primer sets for RT-PCR.**
(PDF)

Table S2 **Primer sets for qPCR.**
(PDF)

Table S3 **Characterization of PSC primary cultures by immunofluorescence staining as demonstrated in Figure S2.** Three independent PSC cultures were analyzed at the second day of culture.
(PDF)
