# Supplementary figures and images for: Stellate Cells from Rat Pancreas Are Stem Cells and Can Contribute to Liver Regeneration
Source: PLoS One. 2012 Dec 13;7(12):e51878. doi: 10.1371/journal.pone.0051878 (PMC3521726; doi:10.1371/journal.pone.0051878)

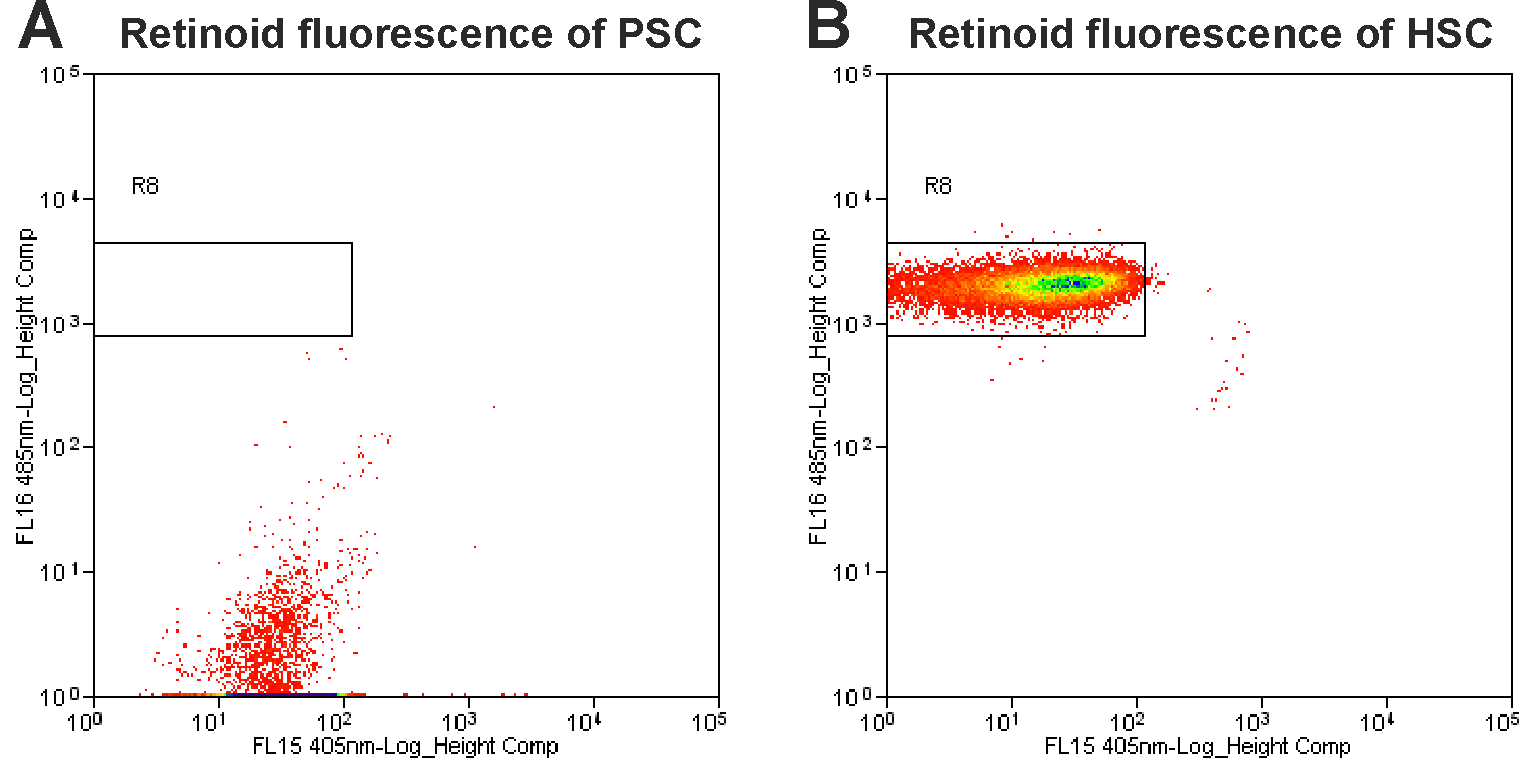

Supplement: Figure S1 — Flow cytometry analysis of retinoid fluorescence in freshly isolated PSC and HSC. (A) PSC and (B) HSC of Wistar rats were analyzed by forward and side scatter with identical settings. Stellate cells with similar morphological properties were excited at 350 nm to measure retinoid fluorescence at 485 nm. In contrast to HSC (gate R8), PSC generally contained low retinoid amounts. The analysis was performed with the flow cytometer MoFlo XDP (Beckman Coulter). (TIF) [file pone.0051878.s001.tif]

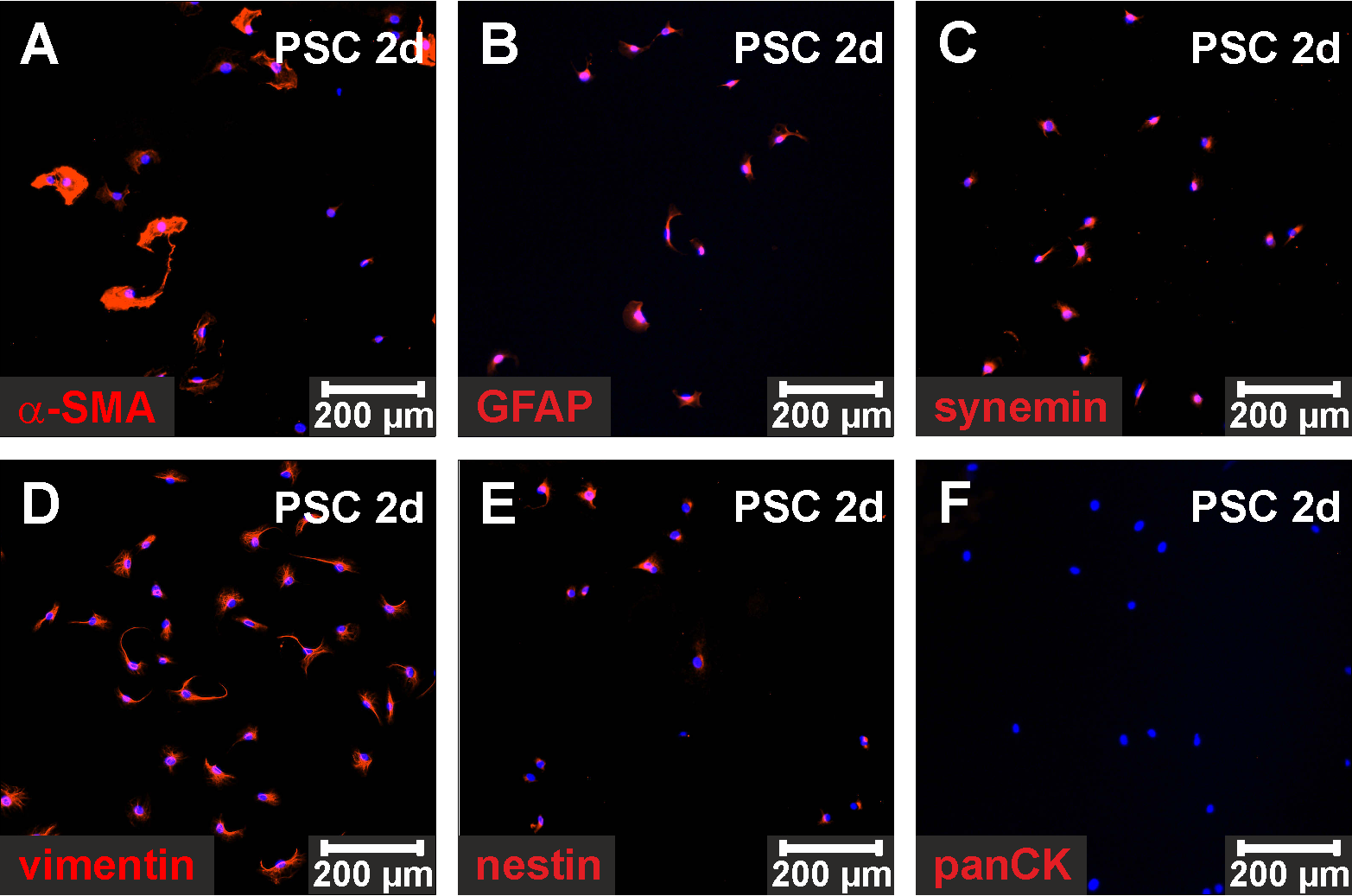

Supplement: Figure S2 — Characterization of PSC by immunofluorescence staining to assess the uniformity of primary cultures. The PSC were analyzed by immunofluorescence at the second day of culture using antibodies against the stellate cell markers (A) α-SMA, (B) GFAP, (C) synemin, (D) vimentin and (E) nestin. These proteins were detected in the vast majority of cells, indicating highly uniform PSC preparations. (F) Antibodies against cytokeratins were used to exclude the pancreatic duct-associated stem/progenitor cells. The number of positive PSC obtained by this method is presented in Table S3. (TIF) [file pone.0051878.s002.tif]

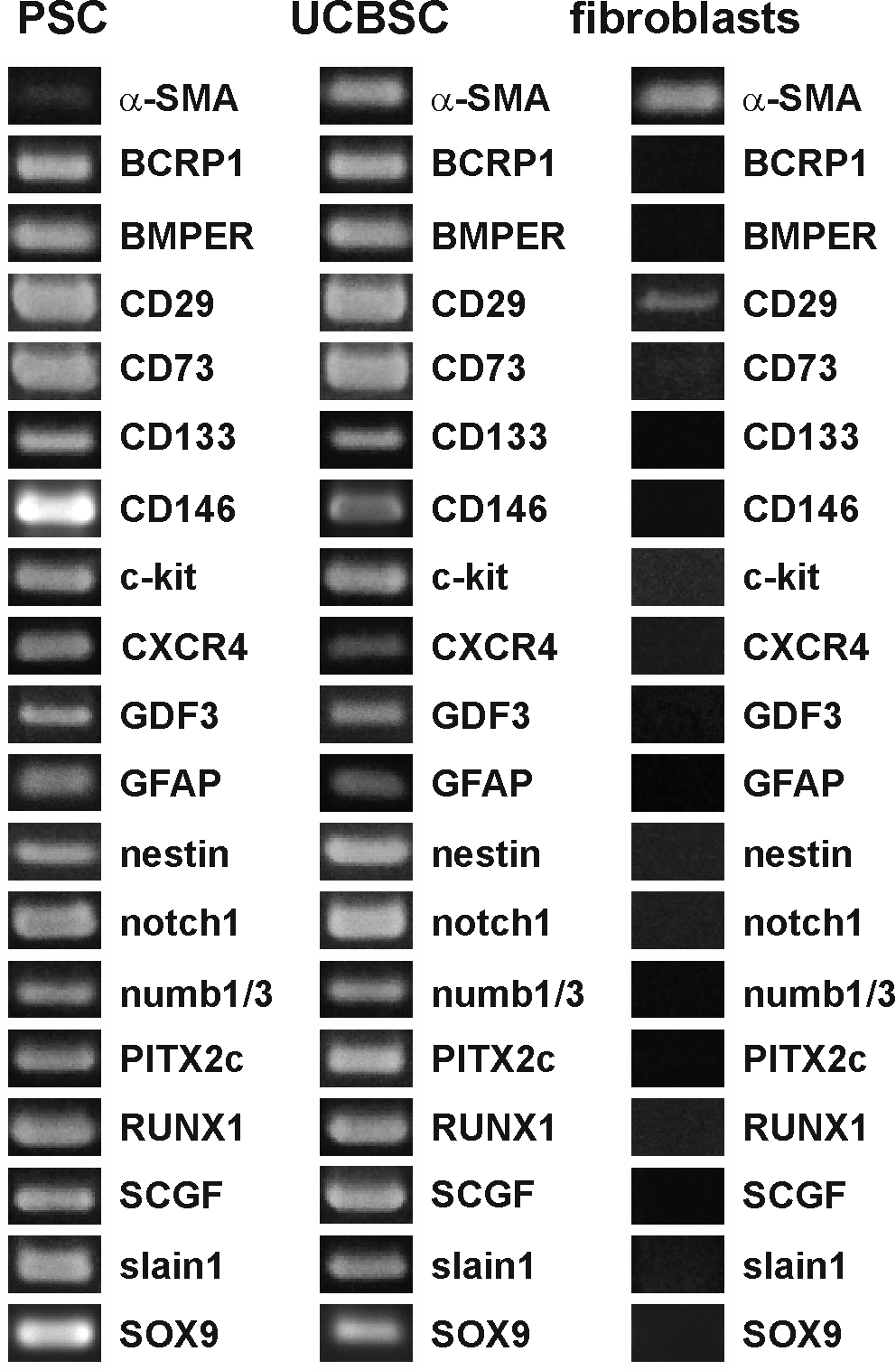

Supplement: Figure S3 — Expression of stem/progenitor cell markers in early primary cultures of PSC. RT-PCR analysis of stem/progenitor cell markers in one-day-cultured PSC, clonally expanded UCBSC and muscle fibroblasts from rats. Freshly isolated PSC and the UCBSC clone 1G11 displayed a similar expression pattern, which was distinct from differentiated cells such as muscle fibroblasts. (TIF) [file pone.0051878.s003.tif]

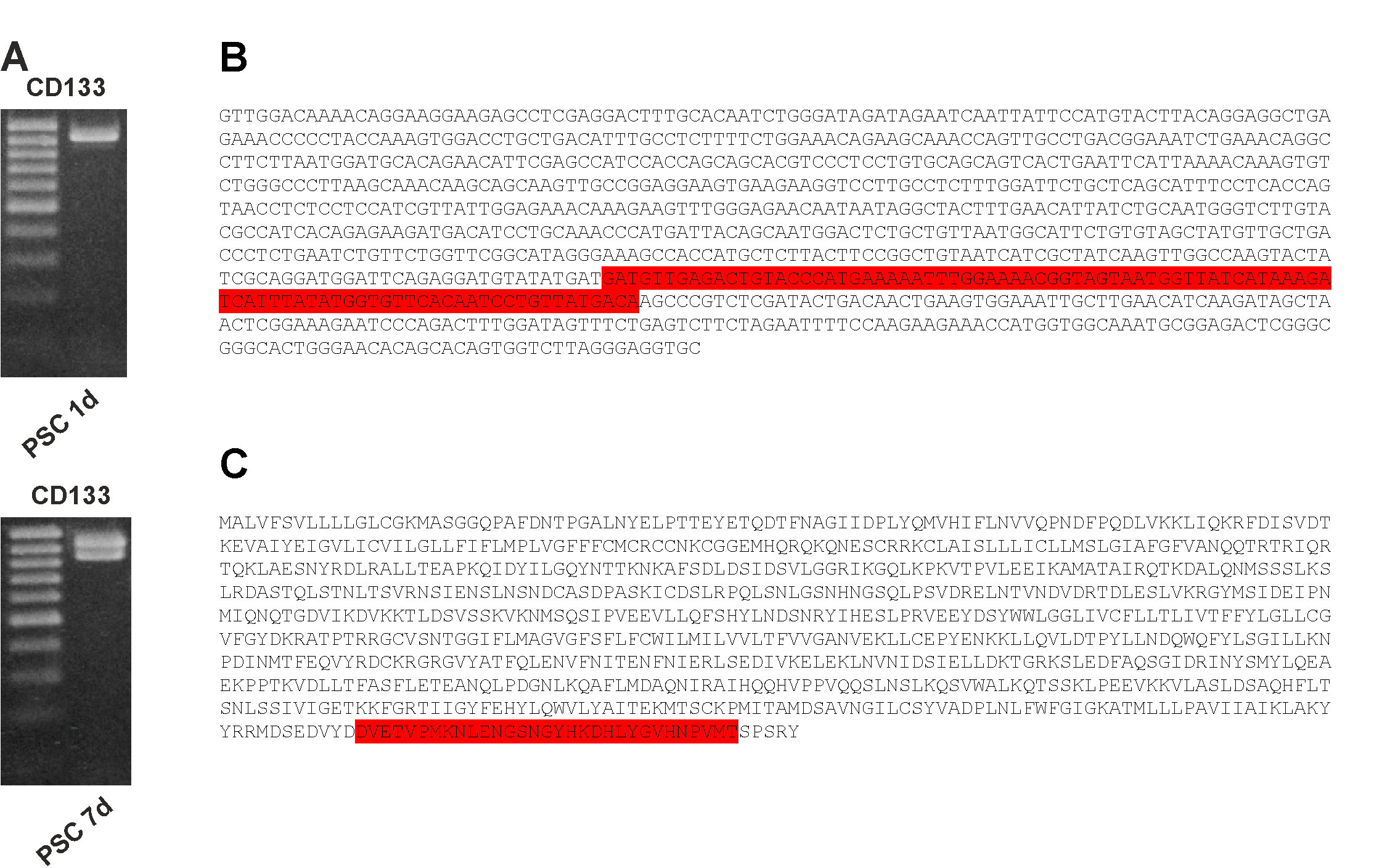

Supplement: Figure S4 — Sequence analysis of CD133 transcript variants in stellate cells at the mRNA and protein level. (A) During culture of PSC two transcript variants of CD133 became detectable by RT-PCR. (B) The shorter transcript variant of CD133 lacks 93 nucleotides (highlighted in red) as investigated by sequencing of the mRNA products obtained by application of the 3′-terminal primers for CD133. This mRNA sequence corresponded to the transcript variant 2 of rat CD133 (gene bank accession number: NM_001110137). (C) The resulting difference of both CD133 transcript variants was highlighted (red) in the complete CD133 amino acid sequence and may explain the appearance of two CD133 protein bands in PSC cultured for 7 days (Fig. 4). (TIF) [file pone.0051878.s004.tif]

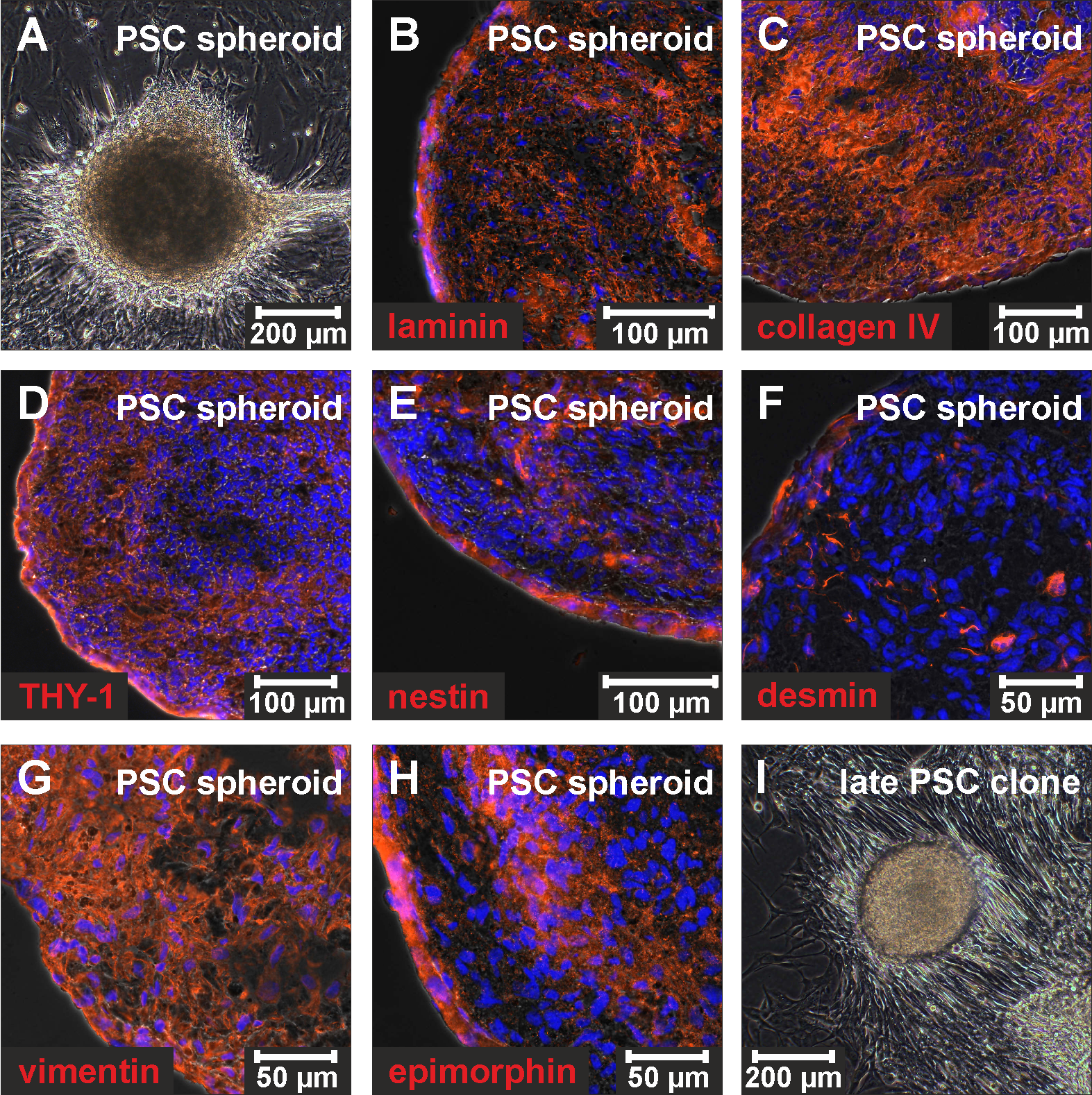

Supplement: Figure S5 — PSC and PSC clones form cell spheroids in vitro . (A) PSC in primary culture formed cell spheroids in the presence of FCS. Cryosections of PSC spheroids were analyzed with antibodies against (B) laminin, (C) collagen type IV, (D) THY-1, (E) nestin, (F) desmin, (G) vimentin and (H) epimorphin (red) after 21 days of culture. The cell nuclei were marked by DAPI (blue). (I) Also clonally expanded PSC (clone 2F5) developed cell spheroids after 14 days of FCS treatment, indicating that spheroid formation is an intrinsic property of stellate cells. (TIF) [file pone.0051878.s005.tif]

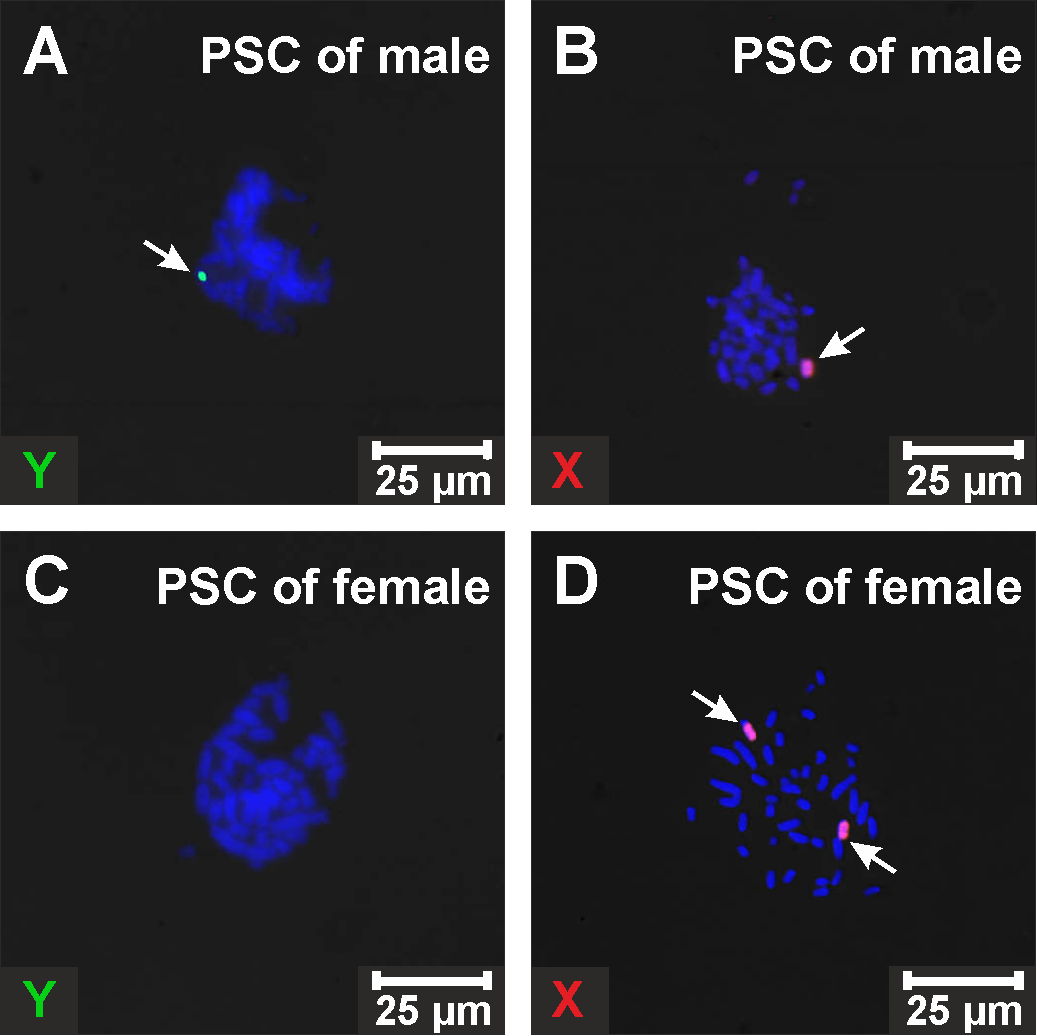

Supplement: Figure S6 — Specificity of gender specific DNA probes. FISH of chromosome Y and X was performed on metaphase spreads of isolated PSC from male and female rats. (A) One chromosome was labeled by chromosome Y probes in male PSC (green; arrow). (B) The chromosome X probe marked a single chromosome in male PSC (red; arrow). (C) Female PSC were without clear labeling after application of the chromosome Y probe (green), (D) but displayed two chromosomes, which were bound by the chromosome X probe (red; arrows). (TIF) [file pone.0051878.s006.tif]
