# Supplementary material for: Stellate Cells from Rat Pancreas Are Stem Cells and Can Contribute to Liver Regeneration
Source: PLoS One. 2012 Dec 13;7(12):e51878. doi: 10.1371/journal.pone.0051878 (PMC3521726; doi:10.1371/journal.pone.0051878)
Supplement: Table S1 — Primer sets for RT-PCR. (PDF) [file pone.0051878.s007.pdf]

Gene	Forward Primer	Reverse Primer	bp	Accession No.
α-FP	CAAACCGGAAGGTTTAACTCTG	ACCATCTTCCCTGTCAGGTCTA	402	NM_012493
α-SMA	TGCTGGACTCTGGAGATG	GTGATCACCTGCCCATC	292	X06801
albumin	GGTCGCAGCTGTCCGTCAGAG	CCACCAAGGACCCACTACAGCAC	878	V01222
β-actin	GCCCTAGACTTCGAGCAAGA	CAGTGAGGCCAGGATAGAGC	390	NM_031144
BCRP1	GCAGTTCAGGTTATGTGGTTCA	GAAACATGAGTTTCCCAGAAGC	444	NM_181381
BDNF	TGGCTGACACTTTTGAGCAC	GCAGCCTTCCTTCGTGTAAC	413	NM_012513
BMPER	TTCGGAGATCCTCACTACAACA	ACCTCCACAAAACTGTCTCCAT	350	NM_001135799
c-kit	TCCGCAAGAATAGACTCGTACA	ATCCCATAGGACCAGACATCAC	350	AF296696
CD29	GAGGAATGTAACACGACTGCTG	ACTTCTTCTGTGAAGCCCAGAG	564	NM_017022
CD73	ACACTCTGGTTTCGAGATGGAT	ATTTCATCTGGGTGTCTGAGGT	480	NM_021576
CD133	TTAATGCAGCACCAGGTACATC	TCGTTGAGCAGGTAGGGAGTAT	370	NM_021751
CD133 3'-terminal	GTTGGACAAAACAGGAAGGAAG	GCACCTCCCTAAGACCACTG	939	NM_021751
CD146	ATCTCTGTGTGGCATCTGTCC	ACTATCACAGCCACGATGACC	487	NM_023983
CX32	ACAGACACGCCTGCATACATT	CACCTTGTGCCTCTTTACCTCT	457	NM_017251
CXCR4	CCTCTGAGGCGTTTGGTG	GCTTCTTCTGGTAACCCATGAC	251	NM_022205
CYP7A1	ATGCCTTCTGTTACCGAGTGAT	CTTCTTCAGAGGCTGCTTTCAT	429	NM_012942
desmin	GACCTAGAGCGCAGAATTGAGT	GCCATCTCATCCTTTAGGTGTC	476	NM_022531
eGFP	AGGACGACGGCAACTACAAG	CTGGGTGCTCAGGTAGTGGT	311	EU056364
GDF3	ACCTGCAGGGTGTGGTTAAG	TGCATGAAGGCATAATTGGA	400	NM_001109671
GFAP	ACATCGAGATCGCCACCTAC	TCCACCGTCTTTACCACGAT	163	L27219
HHEX	TTTCTACATCGACGACATCCTG	CTCCATTTAGCTCTGCGATTCT	486	NM_024385
HNF1α	CCCTACCTGATGGTTGGAGA	TGCTGCAGGTACGACTTGAC	272	NM_012669
HNF4α	GGTCAAGCTACGAGGACAGC	GAGCAGCACATCCTTGAACA	311	NM_022180
HNF6	GCAGGTCAGCAATGGAAGTAAT	CATTTGTCCAGACTCCTCCTTC	498	NM_022671
MRP2	ACTCTTGTCATGATCTGCATGG	TAGCCAGTTCAAGGTTTGTGTG	432	NM_012833
nestin	GAGTGTCGCTTAGAGGTGCAA	TGTCACAGGAGTCTCAAGGGTA	450	NM_012987
notch1	AGAGCTTTTCCTGTGTCTGTCC	CGGTACAGTCAGGTGTGTTGTT	414	NM_001105721
notch3	CCTCTTTCACCTGTACCTGTCC	ACACAGTAGTGGGAGTGGTCCT	496	NM_020087
numb1/3	ATGCACCAATTGAACAGCTTG	GGGCTGAGTCAGTGCCATTA	327	DQ336705
PITX2c exon 4-6	GAGGTGCATACAATCTCCGATAC	AGAGTTGAAGAAGGGGAAGCTC	456	EF519321
RUNX1	GAACCACTCCACTGCCTTTAAC	GTAGGTGTGGTAGCGAGAGGTC	376	NM_017325
SCGF	ACTCTGAAGGCAAAGAGGTCTG	CGTAAGTACCGGCTTAGAGCAT	490	NM_001012459
slain1	CACTGCAGTCAGTAGCAACCTC	GCAGGTTACTCCCATTTATTGC	365	NM_001014139
SOX9	CATCTCTCCTAACGCCATCTTC	GGCAGGTATTGGTCAAACTCAT	306	XM_001081628
