# Supplementary material for: Stellate Cells from Rat Pancreas Are Stem Cells and Can Contribute to Liver Regeneration
Source: PLoS One. 2012 Dec 13;7(12):e51878. doi: 10.1371/journal.pone.0051878 (PMC3521726; doi:10.1371/journal.pone.0051878)
Supplement: Table S2 — Primer sets for qPCR. (PDF) [file pone.0051878.s008.pdf]

Gene	Forward Primer	Reverse Primer	bp	Accession No.
α-SMA	GCACTACCATGTACCCAGGCA	TGCGTTCTGGAGGAGCAATA	102	X06801
β-actin (DNA)	TTAACTTTCCGCCTAGGGTGT	CCAATACTGTGTCCCCTCAAG	188	NC_005111
CD133	TGCTCATGAGTCTTGGCATC	TGTGTTGTATTGCCCCAGAA	164	NM_021751
desmin	AGCCTGGGTCAGAGACAGAA	TATCTCCTGCTCCCACATCC	166	NM_022531
eGFP (DNA)	GGGCACAAGCTGGAGTACAA	GGGTGTTCTGCTGGTAGTGGT	148	EU056364
GDF3	ACTTATGCTACGTGAAGGAGCTG	CAGCTGCAGGTAATGGTAGGAC	218	NM_001109671
GFAP	ACATCGAGATCGCCACCTAC	TCCACCGTCTTTACCACGAT	163	L27219
HPRT1	AAGTGTTGGATACAGGCCAGA	GGCTTTGTACTTGGCTTTTCC	145	NM_012583
synemin	CTGGAGGATGAGAAGGATGC	ATCTCCGGATTGCTTTCTCC	143	NM_001134858
procollagen Iα2	ACCTCAGGGTGTTCAAGGTG	CGGATTCCAATAGGACCAGA	222	NM_053356
RPS6	GGAAGCGCAAGTCTGTCCGA	AGGTCCCAACCGACGAGGCA	130	NM_017160
slain1	GATCGGTATAGCCTGGAGGAC	AAGGGAAATACTGGGAACTGG	160	NM_001014139
SRY (DNA)	AGCCTCATCGAAGGGTTAAAG	GAGGACTGGTGTGCAGCTCTA	151	AF274872
