# Supplementary material for: Stellate Cells from Rat Pancreas Are Stem Cells and Can Contribute to Liver Regeneration
Source: PLoS One. 2012 Dec 13;7(12):e51878. doi: 10.1371/journal.pone.0051878 (PMC3521726; doi:10.1371/journal.pone.0051878)
Supplement: Table S3 — Characterization of PSC primary cultures by immunofluorescence staining as demonstrated in Figure S2. Three independent PSC cultures were analyzed at the second day of culture. (PDF) [file pone.0051878.s009.pdf]

Protein	Positive PSC (isolation 1)	Positive PSC (isolation 2)	Positive PSC (isolation 3)	Positive PSC (mean)	Antibodies (order number / company)
α-SMA	88 ± 1%	96 ± 4%	99 ± 1%	94 ± 3%	IR611, Dako
GFAP	100 ± 0%	91 ± 1%	96 ± 2%	96 ± 3%	ab7779, Abcam
synemin	100 ± 0%	96 ± 1%	95 ± 3%	97 ± 1%	S 9075, Sigma
vimentin	92 ± 2%	96 ± 2%	100 ± 0%	96 ± 2%	M0725, Dako
nestin	100 ± 0%	91 ± 2%	96 ± 2%	96 ± 2%	sc-33677, Santa Cruz Biotech.
panCK	0 ± 0%	0 ± 0%	0 ± 0%	0 ± 0%	CM162 A,B,C, Biocare
